# The Rationality of Science and the Inevitability of Defining Prior Beliefs in Empirical Research

**DOI:** 10.3389/fpsyg.2019.01866

**Published:** 2019-08-13

**Authors:** Ulrich Dettweiler

**Affiliations:** Department of Cultural Studies and Languages, Faculty of Arts and Education, University of Stavanger, Stavanger, Norway

**Keywords:** Bayesian statistics, frequentist statistics, epistemology, prior probability function, rationality of science, philosophy of science

## Introduction

The recent “campaign” in Nature against the concept of “significance testing” (Amrhein et al., 2019), with more than 800 supporting signatories of leading scientists, can be considered as an important milestone and somewhat resounding event in the long on-ongoing struggle and somewhat “silent revolution” (Rodgers, 2010) in statistics over logical, epistemological, and praxeological aspects (Meehl, 1997; Sprenger and Hartmann, 2019), criticizing over-simplified and thoughtless statistical analyses which still can be found in overwhelming many publications to-date. So-called frequentists, the Neyman/Pearson and Fisher schools, and those who apply a hybrid scheme of the two schools (Mayo, 1996) or simple Null Hypothesis Testing (NHST), likelihoodists, and Bayesians alike have debated their approaches over the past decades. This finally led to a discourse facilitated by the *American Statistical Association*, resulting in a special issue of The American Statistician (Vol. 73/2019) titled: “Statistical Inference in the 21st century: A World Beyond *p* < 0.05,” with “43 innovative and thought-provoking papers from forward-looking statisticians” (Wasserstein et al., 2019, p. 1). The special issue proposes both new ways to report the importance of research results beyond the arbitrary threshold of a categorical *p*-value, and some guides of conduct: the researcher should accept uncertainty, be thoughtful, open and modest in their claims (Wasserstein et al., 2019). The future will show if those attempts to statistically better supported science beyond significance testing will be echoed in the publications to come.

A corresponding discourse has been led by the Royal Statistical Society, whereby Andrew Gelman's and Christian Hennig's contribution “Beyond subjective and objective in statistics” has been discussed by more than 50 leading statisticians (Gelman and Hennig, 2017). They suggest to stop using the rather vague terms “objectivity” and “subjectivity,” and replace them with “transparency, consensus, impartiality, and correspondence to observable reality” for the former, and “awareness of multiple perspectives and context dependence” for the latter. Together with “stability,” these should “make up a collection of virtues” that they consider “helpful in discussions of statistical foundations and practice” (Gelman and Hennig, 2017, p. 967).

Yet, questioning the very concept of “objectivity” might be quite provocative and absurd to most empirical scientists who hold “objectivity” to be a central property of observables, or at least to be the property of scientific method that produces pure, value-free facts. In this light, it is interesting to note that both strategies for overcoming the “statistical crisis in science” (Gelman and Loken, 2014) focus on the researchers' conduct and employ *moral* categories for the *ontological* and *epistemological* problem of *what* we should *believe*.

In this article, I will stress the importance of epistemic beliefs in science for the methods we employ. For this purpose, I will recall an argument that Hilary Putnam proposed more than 35 years ago in his critique of scientific realism. Putnam's philosophy of science had been discussed by statisticians like Meehl and Cronbach at that time (Fiske and Shweder, 1986), but his ideas have since been overlooked in the above-mentioned discourses. Putnam claims that the concept of rationality, as it is assumed in science, is in fact deeply irrational, if it considers methods to be purely formal, distinct and free from value-judgements. There is also an informal part inherent to rationality in science which depends on the changing beliefs of scientists.

At the core of Putnam's argument lies a fundamental critique of verficationalism with its correspondence theory of truth, which is disguised in the assumption that there are such things as “objective” facts, independent of our “subjective” experiences, thoughts, and language.

## The Impact of Science on Modern Conceptions of Rationality

A prominent account of such scientific realism can be found in a later work of John Searle, with whom Putnam fought many philosophical battles (Horowitz, 1996; Cruickshank, 2003).

According to Searle, modern science recurs to “default positions” that are not questioned and “any departure from them requires a conscious effort and a convincing argument.” The most central default position implicit in standard empirical research is that we have direct perceptual access to the world through our senses and that the world exists independently of human observation, which is labeled a “correspondence theory of truth” (Searle, 1999).

Yet, the philosophical cost of such an epistemological stance is high: The underlying ontological assumptions in correspondence theories become increasingly counterintuitive and less understandable with the attenuation of their metaphysical ingredients, requiring ability to position the researcher as having an entirely external “god's eye point of view” (Putnam, 1981, p. 49). In other words: Despite the anti-transcendentalist claim of such positivist sciences, the forms of rationality employed derive upon much more substantial metaphysical assumptions than pragmatist methodologies; yet from increased skepticism, the comprehensibility and commonsensical acceptability of science decreases (Dettweiler, 2015).

Despite Putnam has changed his philosophical ideas throughout his life, one constant theme (at least since the 1970ies) is his pragmatist ontological position, which at many points is neither realistic nor ideal. In his claims that, although the world may be *causally* independent of the human mind, the structure of the world (both in terms of individuals and categories) is a function of the human mind and hence is not *ontologically* independent (cf. Brown, 1988). Hereby, Putnam refers to Kant's concept of the dependence of our knowledge of the world on the “categories of thought” and he claims that there is “*a fact of the matter* as to whether the statements people make are warranted or not” (Putnam, 1981, p. 21, cursive by U.D.). This material, realistic reference allows Putnam to talk about warranted truth that is “independent of whether the majority of one's cultural peers would say it is warranted or unwarranted” (ibid). In this respect, Putnam is more than a mere consensus theorist, but not yet a naturalistic realist. He argues instead that “reason can't be naturalized” (Putnam, 1983) and that here and now “truth is independent of justification…, but not independent of *all* justification. To claim a statement is true is to claim it could be justified” (Putnam, 1981, p. 56). Or as Cronbach (1986) reframes Putnam: “Realism is an empirical hypothesis … that can be defended if we observe that a science converges (p. 90).

So, the main challenge to empirical science is the implicit refutation of the claim that the world is accessible independently from the interpretation through our senses and language. It is, according to Putnam, conceptually impossible “to draw a sharp line between the content of science and the method of science,” and “the method of science in fact changes constantly as the content of science changes” (Putnam, 1981, p. 191).

## “Tuning-free” Does not Mean “Value-free”

This has, or rather should have, direct implications to the understanding of modern science and the statistical framework it is built on. Putnam argues that any scientific methodology needs to take into account the prior beliefs of scientists and the degree of uncertainty of hypotheses. This means, on the other hand, that we scientists need to make explicit those beliefs that are implicit in the methodologies we apply and quantify in some way uncertainty.

This is often an alien thought to scientists who apply frequentist statistics in their data analyses and reject the “use of subjective uncertainty in the context of scientific inquiry” (Sprenger, 2016, p. 382). It is the very idea of frequentist statistics, that in the long run, the underlying procedure leads to a (probably) correct result irrespective of the researchers' beliefs. Yet the convenience of standard statistical programs with its many default settings should not disguise the many choices implicitly made in the simplest statistical operations. Most researchers hardly question why we fit the data into a Gausian model with a uniform distribution on the infinite range for each of the parameters, and a uniform distribution for the error term as well? With the decision to model the data linearly, according to a normal function within an infinite range of possible values, there are already a number of value-driven presuppositions in the model before we even have started entering the data. The rationale behind the uniform prior probability functions used in standard statistical models is, of course, that it contains as little information as possible, in order to make it a “neutral” procedure. But as Gelman and Hennig (2017) argue, “even using ‘no need for tuning' as a criterion for method selection or prioritizing bias, for example, or mean-squared error, is a subjective decision” (p. 971).

There is, as Gelman and Hill (2007) state, nothing wrong with modeling data with uniform distributions on all the parameters. They call those models “reference” models, which provide some important preliminary information in a given data analysis. However, “neutral” does not mean “value-free.” We can conceive of many other distribution functions, with more specified parameters, informed by previous research and representing the researchers' prior beliefs, which might better fit the data.

Bayes theorem does provide us with a statistical framework that tells us how data should change our (subjective) degrees of belief in a hypothesis, within a formal model of rational belief provided by the probability calculus. Bayes theorem states that the posterior distribution, i.e., the probability of the parameters given the data, is proportional to the likelihood, which is the probability of observing the data given the parameters (unknowns) multiplied by the prior probability, which represents external knowledge about the parameters.

In fact, Putnam sees subjective Bayesianism as the statistical framework that can assume a formalized language of science in which reliable observations together with some hypotheses can be rationally expressed.

It is from this point that Gelman and Hennig (2017) initiate their proposal to collapse the dichotomy of objectivity and subjectivity altogether. They demonstrate that those prior probability functions are not so much “subjective degrees of belief” but rather “external information” on a specific research question including “restrictions such as smoothness or sparsity that serve to regularize estimates in high dimensional settings, … the choice of the functional form in a regression model, … and … numerical information about particular parameters in a model.” This is why Sprenger (2018) argues that the so-called “subjective Bayesianism” should in fact be understood as “objective,” thereby defending the language of “objectivity” in science.

## Good Science Is a Matter of Ethics, But not Alone

I agree with Gelman and Hennig that the dichotomy of “subjective” and “objective” causes a lot of confusion in science, especially when it is applied to classify statistical methodology. It is misleading to (dis)qualify Bayesian statistics as “subjective” when prior probability functions for each parameter in a model are defined with great rigor and transparency. It is also misleading when frequentist researchers use default settings in analyses and claim “objectivity” on their side.

This is, however, not so much a question of ethics. Nor can this tension be solved with introducing rules for the virtuous scientist. It is rather a symptom of a fundamental *epistemological* crisis in modern science. The philosophy of science has been too detached from the empirical sciences and statistics for too long, and those gaps need to be bridged with the education of scientists in epistemology, a claim made by Meehl more than 20 years ago (Meehl, 1997). The enhanced rigor of the scientific enquiry will then follow, since the scientific virtues are inspired by the epistemic beliefs that scientists hold. We simply need to learn again to argue for our epistemological stances, and to define the epistemic claims we make with our statistical analyses, given the data. The epistemologically informed scientist would certainly not be scared to endorse subjectivity as a reliable philosophical concept for empirical science, as Putnam has shown.

Or, as Ian Hacking wittingly summarizes this crisis, all we need to do is think harder, not more objectively (Hacking, 2015).

## Author Contributions

The author confirms being the sole contributor of this work and has approved it for publication.

### Conflict of Interest Statement

The author declares that the research was conducted in the absence of any commercial or financial relationships that could be construed as a potential conflict of interest.
